# Curcumin-Loaded Liposomes in Gel Protect the Skin of Mice against Oxidative Stress from Photodamage Induced by UV Irradiation

**DOI:** 10.3390/gels10090596

**Published:** 2024-09-16

**Authors:** Yongli Zhang, Bin Sun, Lu Wang, Wang Shen, Si Shen, Xiaoman Cheng, Xuan Liu, Hongmei Xia

**Affiliations:** College of Pharmacy, Anhui University of Chinese Medicine, Hefei 230012, China; 18119742560@163.com (Y.Z.); 13721231091@163.com (B.S.); 15155063158@163.com (L.W.); 15155034624@163.com (W.S.); shensi@stu.ahtcm.edu.cn (S.S.); xmcheng@stu.ahtcm.edu.cn (X.C.); liuxuan09131005@163.com (X.L.)

**Keywords:** curcumin, liposomes in gel, photodamage, antioxidant

## Abstract

Prolonged exposure to ultraviolet (UV) irradiation can cause oxidative stress in the skin, accompanied by rapid immunosuppressive effects, resulting in a peroxidation reaction throughout the body. Curcumin (Cur), as the bioactive compound of turmeric, is a natural polyphenol with potent antioxidant properties but is often overlooked due to its poor solubility and low bioavailability. In this study, curcumin-loaded liposomes in a sodium alginate gel complex preparation were designed to improve the bioavailability of curcumin and to study its preventive effect on photodamage. Cur-loaded liposomes (Cur-L), Cur-loaded gel (Cur-G) based on an alginate matrix, and curcumin-loaded liposomes in gel (Cur-LG) were prepared, and their antioxidant effects and drug diffusion abilities were evaluated. The antioxidant capacity of Cur, Cur-L, Cur-G, and Cur-LG was also studied in a mouse model of photodamage. Cur had the highest antioxidant activity at about 4 mg/mL. Cur-LG at this concentration showed antioxidant effects during 1,1-diphenyl-2-trinitrophenylhydrazine (DPPH) and H_2_O_2_ experiments. During the UV light damage test, Cur-LG demonstrated the ability to effectively neutralize free radicals generated as a result of lipid peroxidation in the skin, serum, and liver, thereby enhancing the overall activity of superoxide dismutase (SOD). In conclusion, using Cur-LG may protect against epidermal and cellular abnormalities induced by UV irradiation.

## 1. Introduction

The main types of UV radiation affecting the body’s skin include ultraviolet A (UVA) and ultraviolet B (UVB). Among these, UVA (320–400 nm) can be absorbed by DNA, induce the indirect formation of DNA photoproducts, and lead to changes in other macromolecules [1]. It may directly affect local immune cells because of the oxidative free radicals released. UVB (280–320 nm) can penetrate the basal layer of human skin [2] and is reflected, absorbed, and transmitted by complex skin structures and chromophores such as melanin, bilirubin, and hemoglobin [3]. Other cellular components such as DNA, cell membranes, and vitamin D can also absorb UVB rays [4]. If not repaired, the DNA damage caused by ultraviolet radiation may lead to mutations in oncogenes and tumor suppressor genes, leading to skin cancer. UVB mainly reaches into the epidermis layer and induces changes in epidermal cells at the molecular level directly. Both types of radiation stimulate skin cells to produce signals that result in rapid or slow responses at the local and systemic levels mediated by the skin’s neuroendocrine system [5].

Skin exposure to UVA or UVB radiation interferes with the production and removal reactive oxygen species, leading to oxidative stress; affecting the oxidative modification of nucleic acids, proteins, and lipids; and hindering efficient cell metabolism and normal skin function [6]. On the other hand, the UV irradiation process can produce proapoptotic proteins, induce lipid peroxidation, and affect related signaling molecules. All these molecules can cause proinflammatory reactions in adjacent cells throughout the body. So far, the harmful oxidation effect of UV radiation on skin tissues has been confirmed in mouse liver studies. A significant increase in triglycerides was accompanied by a decrease in glutathione, and the activities of glutathione reductase, superoxide dismutase, and catalase were also affected [7]. In addition, the level of glutamic acid in the blood of mice treated with UV irradiation was increased, and these substances were transported to the brain and significantly affected synapses. Systematic human studies have shown that UV radiation reduces blood pressure by increasing nitric oxide production in the skin [8,9].

In recent years, the medical costs of treating bone-related trauma, infections, and tumors have been increasing [10]. Currently, clinical treatments for bone injury (such as autografts, allografts, and xenografts) are not widely available due to the potential risks of disease transmission, infection, and host rejection. Bone tissue engineering (BTE), a new method of using scaffolds to inoculate cells or incorporate bioactive growth factors to promote bone repair and regeneration, is considered to be able to avoid the above problems and provide an innovative platform for regenerative medicine [11]. Hydrogels are polymer scaffolds that have several potential advantages in bone repair [12]. Turmeric belongs to the ginger family and is widely used in the food industry as a pigment, seasoning, and preservative agent due to its natural color and odor. The essential oil of turmeric, with a unique smell, is mainly composed of monoterpenes, sesquiterpene, alcohols, and carbonyl compounds [13]. Turmeric is also a famous natural medicinal plant, and curcumin, as its main active ingredient, has been widely studied for its anti-inflammatory, antioxidant, antibacterial, and anticancer properties. Curcumin may specifically disrupt the nuclear factor kappa B (NF-κB) pathway, target cancer stem cells, and inhibit histone deacetylase signaling to treat cancer patients with poor prognoses [14]. The inhibition of NF-κB activation by curcumin combined with the reduced downstream induction of intercellular adhesion molecule-1, cyclo-oxygenase-2, and macrophage chemotactic protein-1 significantly reduced methionine- and choline-induced liver inflammation [15]. However, curcumin is characterized by low solubility, poor stability, and non-specificity in practice [16]. It is readily converted into compounds such as glucuronic and sulfonic acid in the intestine. The rapid metabolism and short half-life of curcumin result in low bioavailability. In recent years, several methods have emerged to improve the bioavailability of curcumin. Ethyl polymethacrylate nano-capsules loaded with curcumin in lamb diets enhanced the absorption of curcumin at the intestinal level and effectively improved the growth of lambs [17]. When curcumin was combined with N-n-butyl haloperidol iodide to form a new combination, curcumin’s availability and antitumor effect were significantly increased [18].

Sodium alginate (SA) is a polysaccharide found in seaweed and is naturally anionic. Due to its biocompatibility, low toxicity, and relatively low cost, it has been widely used in the food, pharmaceutical, and medical device industries. Alginate can form three-dimensional cross-linked networks by various methods under mild gelation conditions. Alginate gels are typically nanoporous (pore size ~5 nm) [19] and can allow for small molecules to rapidly diffuse in the gel, shrink or expand under specific stimulation (temperature or pH, etc.), and release drugs. The protonation of the carboxylate in the alginate structure leads to a change in viscosity, taking on a complex texture in acidic media and having a rubbery taste [20]. Alginate can absorb wound fluid, convert it into a gel, and then supply water to the dry wound to maintain a physiologically wet microenvironment, thus effectively reducing bacterial infection in the wound [20]. The injection of alginate gel into ischemic muscle tissue could maintain the long-term release capacity of intravascular growth factors and promote the formation and maturation of blood vessels [21,22].

Therefore, the different concentration of Cur, Cur-L, Cur-G, and Cur-LG was prepared, and their ability of free radical scavenging and diffusion capacities were studied. A mouse photoaging model was established to study the inhibiting ability of Cur and its preparations against UV damage and their suppressive effect on oxidative stress responses in serum, spleen, and liver tissues.

## 2. Results and Discussion

### 2.1. Antioxidant Capacity of Curcumin-Loaded Liposomes in Gel on H_2_O_2_

**Figure 1:**
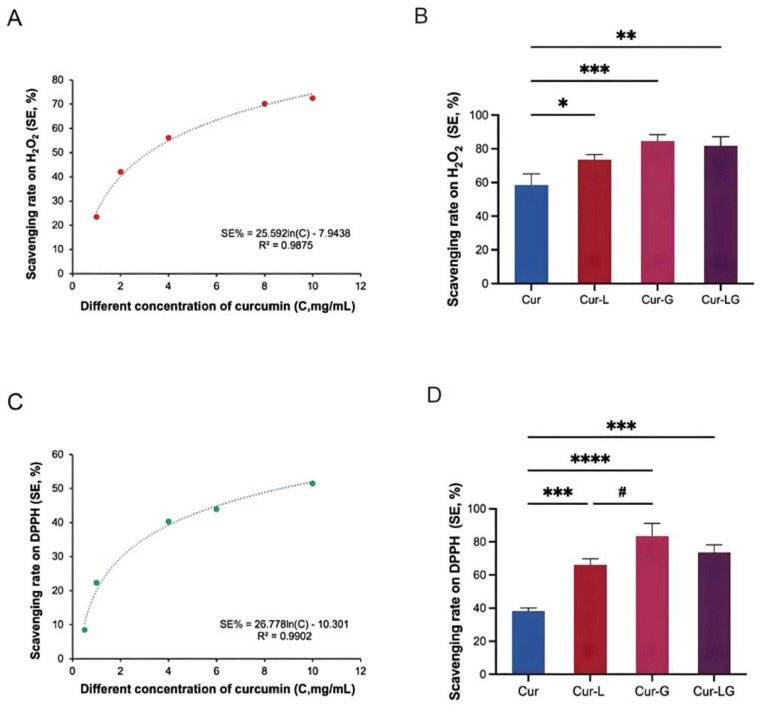
Scavenging capacity on H_2_O_2_ and DPPH. (**A**) Clearance of H_2_O_2_ by curcumin solution with different concentrations; (**B**) the scavenging ability of Cur, Cur-L, Cur-G, and Cur-LG on H_2_O_2_; (**C**) the relationship between curcumin solution and DPPH scavenging rate. (**D**) The scavenging ability of Cur, Cur-L, Cur-G, and Cur-LG on DPPH; * *p* < 0.05, ** *p* < 0.01, *** *p* < 0.001, **** *p* < 0.0001, # *p* < 0.05.

The main sources of free radicals are the normal physiological and the metabolic productions of chemical toxins in the body. The O-O bond of H_2_O_2_ in the molecular structure can easily be broken to form H-O free radicals [23]. When free radicals increase in the body, they will damage the chemical structure of essential biological macromolecules. When they exist in skin tissue, it can cause skin peroxidation, induce inflammatory reactions, lead to the loss of collagen in the skin, and accelerate the skin’s aging process [24]. Therefore, the antioxidant activity of curcumin was evaluated by its action of scavenging free radicals. According to Figure 1A, curcumin’s inhibition of free radicals showed an increasing trend with increasing concentrations (0.5, 0.8, 1, 2, 4, and 6 mg/mL). In Figure 1B, the inhibition rates of free radicals by Cur, Cur-L, Cur-G, and Cur-LG were 58.47 ± 6.67%, 73.43 ± 3.23%, 84.5 ± 3.9%, and 81.63 ± 5.45%, respectively.

### 2.2. Scavenging DPPH Free Radical Experiment

DPPH is a very stable nitrogen-centered free radical. The conjugation of three benzene rings prevents the unpaired electrons on the nitrogen atom sandwiched between them from functioning as an electron pair [25]. In the presence of a free radical scavenger, electrons are added to the DPPH free radical, resulting in a lightening of color. The degree of color fading is quantitatively related to the number of electrons received and can be measured spectrophotometrically. Therefore, DPPH can be used to evaluate the antioxidant activity of Cur, Cur-L, Cur-G, and Cur-LG. In Figure 1C, curcumin’s scavenging rate of free radicals increased with increasing concentration. The scavenging rates on DPPH free radicals by Cur, Cur-L, Cur-G, and Cur-LG at the same concentration shown in Figure 1D were similar to the results of H_2_O_2_ experiments. The protective effect of sodium alginate gel surrounded curcumin in Cur-G gives full play to its antioxidant effect. Cur-LG had an excellent antioxidant effect when Cur was synergistic with liposomal gel.

### 2.3. Release Rates of Cur, Cur-L, Cur-G, and Cur-LG across Dialysis Membranes

**Figure 2:**
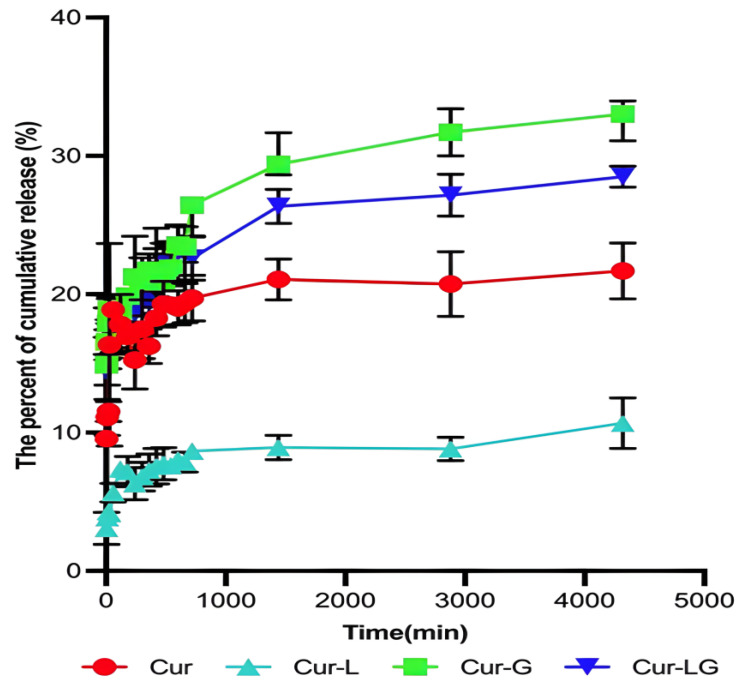
Release rates of Cur, Cur-L, Cur-G, and Cur-LG across dialysis membranes.

In order to protect against UV damage to the skin, the drug must reach and remain in the skin. In the dialysis release profile (Figure 2), the cumulative release rates of Cur, Cur-L, Cur-G, and Cur-LG were 19.76 ± 1.54%, 3.26 ± 3.27%, 33.02 ± 1.91%, and 27.64 ± 2.05% at the time of 4320 min (72 h). Compared with the Cur group, the Cur-LG and Cur-G groups could release large amounts of the drug in a short period and maintain a particular concentration in the middle and late stages because of the osmotic pressure of the gel. The drug release rate of Cur-L was slower and thus could achieve a slow release. Still, the release amount was lower, probably because of the skeletal characteristics of liposomes, and the many functional groups in phospholipids that are sensitive to oxidation and hydrolysis.

### 2.4. Macroscopic Changes

#### 2.4.1. Changes in Skin Surface Inflammation and Proliferation: Subcutaneous Vascularity in Mice

Erythema, epidermal hyperplasia, and dermal vasodilation were observed after the skin was exposed to UV radiation [26]. The main reason for this was that UV radiation on the skin leads to a significant upregulation of proangiogenic and proinflammatory mediators, resulting in excessive vascular permeability [27]. Studies have shown that UV radiation causes pronounced upregulation of vascular endothelial growth factor and downregulation of thrombospondin-1 in epidermal keratinocytes, inducing elastic fiber damage and marked dermal angiogenesis [28].

**Figure 3:**
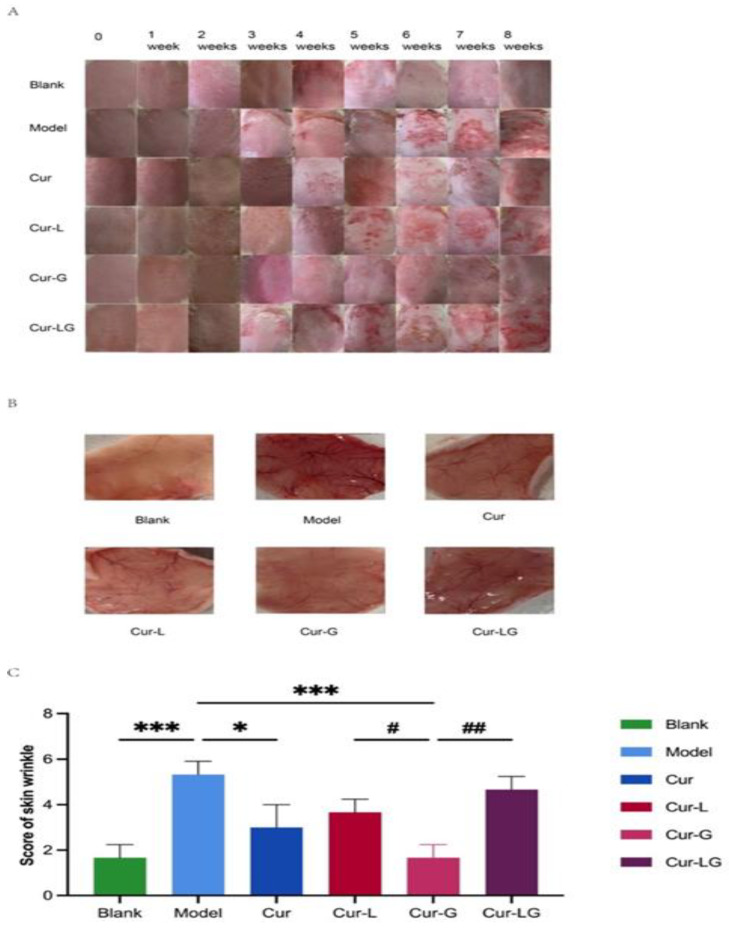
Cur/Cur-L/Cur-G/Cur-LG prevented macroscopic skin changes in mice induced by UV radiation. (**A**) The changes on the back skin surface of mice and subcutaneous tissue maps after eight weeks UV radiation; (**B**) observation of subcutaneous blood vessels of mice at the eighth week; (**C**) visual scoring of mouse dorsal surface skin. * *p* < 0.05, *** *p* < 0.001, # *p* < 0.05, ## *p* < 0.01.

Within eight weeks of UVA and UVB irradiation, compared to the blank group, the model group mice showed significant red scars and skin thickening signs due to dermatitis on their backs starting from the third week. After removing the skin, the blood vessels in the skin of the mice were severely visually dilated, with clear red blood vessels visible (Figure 3A,B). The mice of Cur, Cur-L, Cur-G, and Cur-LG showed different degrees of damage prevention. After UV irradiation, the mice of Cur group appeared dry and scaly, gradually improving after the seventh week. However, skin damage still occurred, and subcutaneous vessels were dilated compared to the blank group. In the fifth week, the mice of the Cur-L group showed significant reddish-brown scab scars; in the eighth week, the scar symptoms decreased. However, skin inflammation was still present, similar to vasodilation, compared to that of the Cur group. The mice of the Cur-G group showed signs of slightly red skin inflammation in the sixth week, a small number of wrinkles in the seventh week, and almost no damage in the eighth week. The vascular condition was nearly the same as that in the blank group. The redness and swelling of the skin of the Cur-LG group mice were more severe due to the drug’s slow release, with scabs starting from the third week and showing apparent dry scales in the sixth week. Subcutaneous vasodilation in the Cur-LG group was less severe than that in the model group.

#### 2.4.2. Score of Mouse Skin Injury

Photos of the skin on the backs of the mice in each group after 8 continuous weeks of UV radiation are shown in Figure 3C. The skin of the mice in the blank group was moist and smooth, while the skin of the mice in the model group was red and inflamed, with large crusts and even a tendency toward developing flesh-colored lesions, which are typical characteristics of photoaging skin. There was a significant difference (*p* < 0.001) in the macroscopic score between the model and the blank groups, indicating that the photoaging model was successfully generated. Compared with the model group, the mice of the Cur and Cur-L groups showed lighter wrinkles and less skin damage. The skin of the mice in the Cur-G group showed slight redness and wrinkles, and some mice had even recovered, exhibiting smooth skin. Their macroscopic scores were significantly lower than those of the mice in the model group (*p* < 0.001), indicating that Cur-G had a specific preventive effect on skin photoaging.

### 2.5. Effects of Cur/Cur-L/Cur-G/Cur-LG on the Spleen Index of Photodamaged Mice

**Figure 4:**
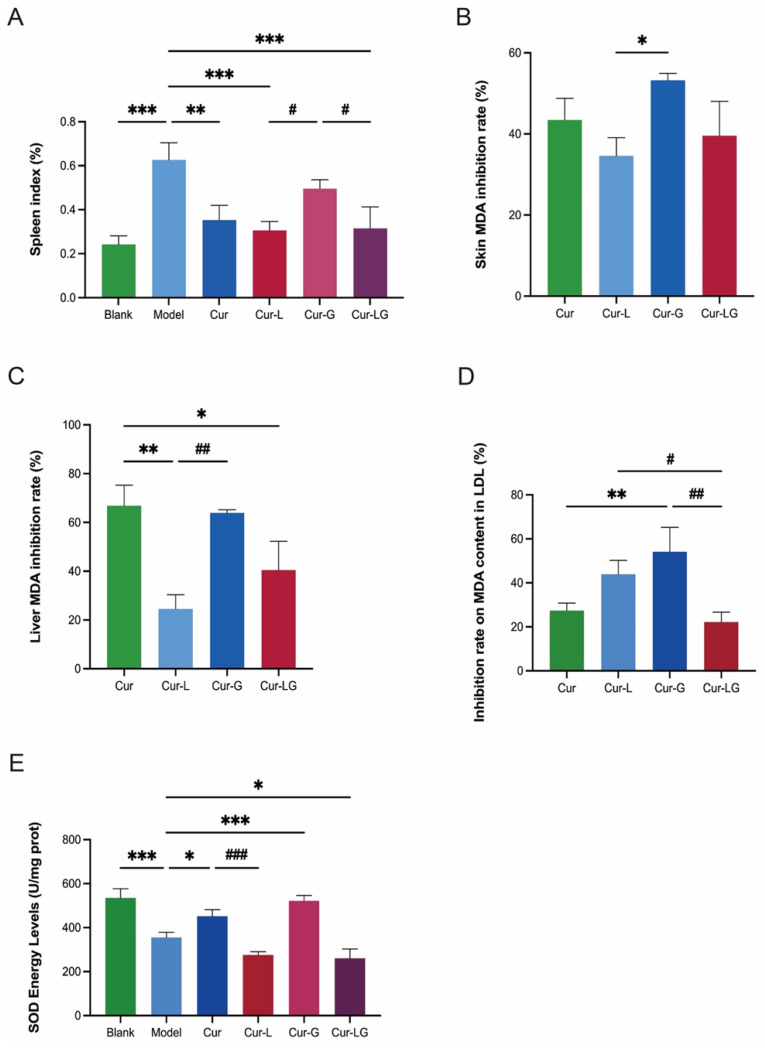
(**A**) Spleen index; (**B**) inhibitory rate of different samples on MDA production in skin; (**C**) inhibitory rate of different samples on MDA production in liver; (**D**) inhibition rate of Cur/Cur-L/Cur-G/Cur-LG on MDA generation in LDL; (**E**) total vitality of SOD in the skin. * *p* < 0.05, ** *p* < 0.01, *** *p* < 0.001, # *p* < 0.05, ## *p* < 0.01, ### *p* < 0.001.

The spleen is the largest immune organ in the body [29]. The strength of immune function was assessed by measuring the weight of the spleen and calculating the spleen index (ratio of spleen to body weight). According to Figure 4A, the spleen index of the model group was 0.63 ± 0.08%, which was higher than that of the blank group at 0.24 ± 0.04%. There was a significant difference (*p* < 0.001), indicating that photodamage caused abnormal systemic immune reaction, resulting in enhanced splenic function and thus splenomegaly. The spleen indices of the Cur, Cur-L, Cur-G, and Cur-LG groups were 0.41 ± 0.12%, 0.34 ± 0.07%, 0.473 ± 0.04%, and 0.315 ± 0.01%, respectively, which were all lower than those of the model group, indicating that there was an inhibitory effect on all hyperimmune reactions. The enlargement in the Cur-G group was higher than that in the Cur-LG group (*p* < 0.05), indicating that liposomes in hydrogel could enhance the protective effect on the spleen.

### 2.6. Effect of Cur/Cur-L/Cur-G/Cur-LG on MDA Content in Skin and Liver Tissue of Photodamaged Mice

UV light induces a large production of reactive oxygen species in the skin. It attacks various biological molecules, of which the peroxidation of polyunsaturated fatty acids results in the production of MDA and other reaction products. Therefore, MDA is widely accepted as a biomarker of oxidative stress, and its measurement can be used as the test indicator of photoaging damage. MDA in skin and liver homogenates were measured and demonstrated the ability of Cur and its different preparations to scavenge MDA. In the skin map (Figure 4B), the MDA clearance of Cur, Cur-L, Cur-G, and Cur-LG was 43.45 ± 5.38%, 34.62 ± 4.46%, 53.21 ± 1.76%, and 39.56 ± 8.51%, respectively. Among them, the MDA clearance rate of Cur-G was higher than that of the Cur-L, with a significant difference (*p* < 0.05). In the graph of MDA clearance in the liver (Figure 4C), the MDA clearance rates of Cur, Cur-L, Cur-G, and Cur-LG were 66.38 ± 8.42%, 24.53 ± 5.83%, 63.94 ± 1.26% and 40.44 ± 11.8%, respectively. The MDA clearance rate of Cur-L was lower than that of Cur, with a significant difference (*p* < 0.01). The MDA clearance rate of Cur-G was similar to that of Cur and there was a significant difference from that of the Cur-L group (*p* < 0.01).

### 2.7. Effect of Cur/Cur-L/Cur-G/Cur-LG on MDA Content in Blood LDL in Photodamaged Mice

LDL in the blood itself does not cause damage to the body. UV-irradiation-induced reactive oxygen species oxidize polyunsaturated side chains to produce MDA, which prevents the standard decomposition of fats in LDL into H_2_O and CO_2_ [30]. The level of MDA was measured in LDL to demonstrate the extent of damage caused by UV irradiation, which was 27.41 ± 2.77% and 54.07 ± 9.13% in the Cur and Cur-G groups; there was a significant difference between the Cur-G and Cur groups (*p* < 0.01). The MDA clearance rates in the Cur-L and Cur-LG groups were 43.92 ± 5.13% and 22.22 ± 3.63%, with no significant differences compared to that of the Cur group (*p* > 0.05) (Figure 4D). The Cur-L and Cur-G groups had higher and significantly different MDA clearance rates than the Cur-LG group. Due to the advantages of liposomes and gel, Cur-LG had slow-release properties [31], resulting in insufficient drug release to remove MDA from LDL in a short period.

### 2.8. Effect of Cur/Cur-L/Cur-G/Cur-LG on Skin SOD Activity in Photodamaged Mice

SOD is an essential enzyme for the elimination of superoxide radicals to protect cells and maintain the body’s immune levels by scavenging free radicals. The superoxide anion is generated via the xanthine and xanthine oxidase reaction system [32], and the superoxide anion reduces azotetrazolium to produce blue methanogen, which absorbs at 560 nm. SOD scavenges the superoxide anion, thereby inhibiting the formation of methanogen, and the color change can be measured by UV spectrophotometry. In Figure 4E, one can see that the total SOD activity in the positive control group was 355.82 ± 23.32 U/mg prot, which is significantly different from that of the blank group (*p* < 0.001), demonstrating that UV inhibited the activity of SOD in the skin tissue. The SOD content in the Cur group was 452.2 ± 29.86 U/mg prot, which is significantly different from that of the positive control group (*p* < 0.05); the total activity in the Cur-G group was 521.61 ± 24.65 U/mg prot. The total SOD activities in the Cur and Cur-G groups were higher and had a preventive effect on UV-induced oxidative stress. This group did not show significant difference (*p* > 0.05), indicating that the Cur-L and Cur-LG groups had a better slow-release effect.

### 2.9. Discussion

It was shown that ultraviolet radiation could cause abnormal levels of free radicals in the skin and other issues of the body, leading to oxidative stress reactions. Experiments had demonstrated that Cur, Cur-L, Cur-G, and Cur-LG could effectively prevent damage caused by UV radiation. The dialysis and mouse antioxidant experiments indicated that Cur-L and Cur-LG had good sustained-release properties.

Curcumin has a broad therapeutic potential and is one of the most valuable natural products. Phenol hydroxyl and β-diketone is the leading active group of curcumin [33], which can eliminate free radicals and achieve antioxidant effects. The methoxy group on the benzene ring on both sides of the molecular structure can enhance its antioxidant activity. DPPH is a stable nitrogen-centered chromogenic free radical that produces characteristic absorption peaks at a wavelength of 517 nm [34]. The antioxidant activity of curcumin at different concentrations depends on its ability to donate electrons. In in vitro experiments, the 4 mg/mL curcumin solution had the most vital ability to reduce free radicals. It has the most potent antioxidant ability in the DPPH and H_2_O_2_ antioxidant activity experiments.

However, curcumin is yet to be widely used in clinical practice because it is a hydrophobic molecule and is insoluble in water [35]. The phenolic hydroxyl group in curcumin, which is sensitive and unstable in alkaline solution, is ionized into phenol oxygen negative ions under alkaline conditions, making curcumin lose the dominant characteristic of the phenolic hydroxyl group [6]. Therefore, curcumin is very easy to deactivate in the human body and has a short half-life as a drug. Curcumin has a weak net negative charge, and the conformation of the β-diketone group in its structure is highly dependent on its intermolecular bonding behavior [36]. Therefore, it is necessary to develop appropriate dosage forms to increase the utilization rate of curcumin.

Liposomes, which are mainly composed of phospholipids and cholesterol, have the same bilayer structure as the cell membrane of the body, such as skin, which is easy to fuse with the cell membrane [37]. Liposomes entering the body can be swallowed by macrophages as foreign bodies and concentrated in liver, spleen, lymphocytes, macrophages, and other cell organs, which have an excellent targeting effect. However, the stability of liposomes is poor, and external factors easily disrupt the lipid bilayer [38].

**Figure 5:**
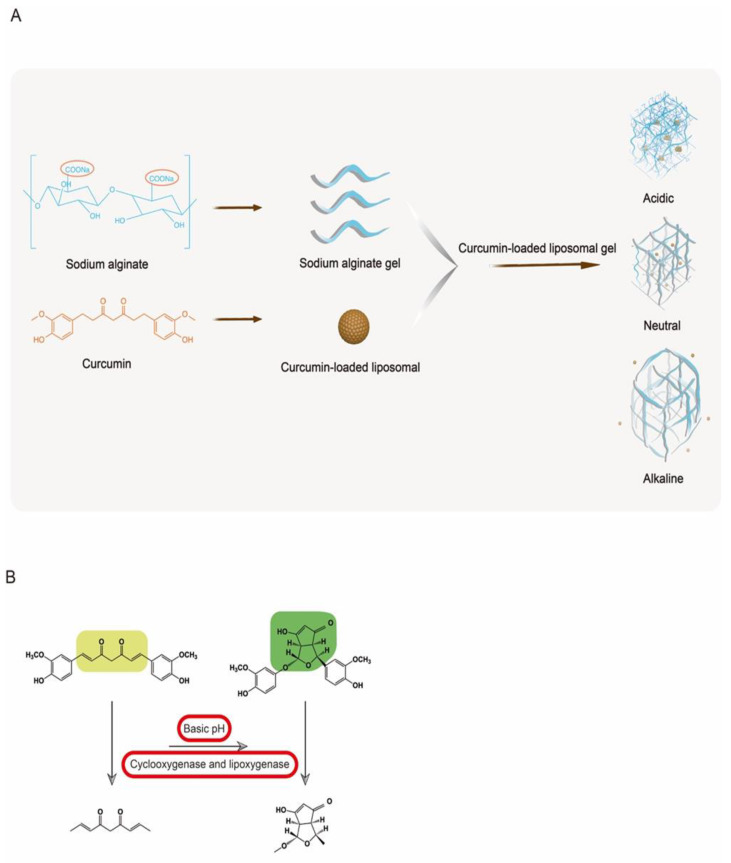
(**A**) The relationship between Cur-LG stability and pH; (**B**) under alkaline pH or the action of catalytic enzymes such as cyclooxygenase and lipoxygenase, curcumin can undergo intramolecular cyclization of phenoxy radical intermediates generated by autoxidation or free radical mediation.

In the experiment, sodium alginate was used to prepare the gel, which could maintain the activity of wrapping drugs, proteins, and other active substances, thus improving the medical applicability of curcumin [39,40,41]. The viscosity, pH value, and cross-linking form of sodium alginate are all factors affecting drug release [42]. According to the experimental characteristics and equipment, the size of molecules and the amount of penetration that could pass across the dialysis membrane. Sodium alginate is a polymer with basic groups, and hydrogels made from it have pH-responsive behavior. As shown in Figure 5A, under acidic conditions, the -COO- group is protonated into the -COOH group and strong hydrogen bonds are formed between -COOH, -OH, and -NH_2_. The hydrogen bonds make the gel structure less mobile and allow for more stable coalescence and adsorption of the drug; under neutral conditions, the -COOH group on the alginate starts to deprotonate; in an alkaline environment (pH 7.4), due to massive deprotonation, the dissociation of most of the hydrogen bonds in the lattice caused electrostatic repulsion, leading to the expansion of the lattice structure and the release of the drug [43,44]. However, high pH can dissociate the hydrogel. In addition, the three-dimensional spatial arrangement of the gel is spatially supported, and the encapsulated drug is not susceptible to spatial movement. It is also widely used to treat skin diseases due to its advantages, i.e., it is non-toxic and non-irritant [45].

Due to the instability of liposomes, Cur-L might precipitate and result in a weakened ability to penetrate the dialysis membranes. By dispersing the liposomes in the gel, the network structure of the gel provides spatial support for the liposomes, which improves the stability of the liposomes and enables them to give full play to their slow-release properties. The same concentration of Cur-L/Cur-G/Cur-LG was designed and prepared according to the characteristics of the liposomes and gel to study their preventive effects on photodamaged mice.

The MDA indicator, measured in this study, is generated during the metabolism of oxygen free radicals. MDA can affect polymers such as nucleic acids and proteins, leading to a series of oxidative damage and accelerating the rate of body damage [46]. Long-term UV radiation would cause degeneration of elastic tissue in the upper dermis, thickening of the epidermis, reduction in premature collagen fibers in the dermis, proliferation of elastic fibers, and increase in reticular fiber. In addition, UV radiation can cause lipid peroxidation in the skin, leading to an abnormal increase in MDA content. In antioxidant experiments, Cur-G could remove MDA from tissues more quickly than Cur-L and Cur-LG with the sustained release effect. Studies have shown that intact skin is naturally acidic, with a pH between 4 and 6. When the skin is damaged, the dermis is disrupted, exposing internal tissue and interstitial fluid, increasing the pH of the wounded surface, the pH of the wound increases, and approaching the physiological pH that favors bacterial infection (pH 7.4) [47], which in turn leads to an increase in skin pH between 7.5 and 8.9, causing inflammation and resulting in a prolonged wound healing time [48]. Curcumin can undergo intramolecular cyclization by autoxidation or free-radical-mediated generation of phenoxy radical intermediates at alkaline pH or by catalytic enzymes such as cyclooxygenase and lipoxygenase; this leads to a long chain reaction of the conjugated β-endione chain segment between the two benzene rings to produce intramolecular carboxylation products with tetrahydrofuran and cyclopentadiene structures [6-hydroxy-1-(4-hydroxy-3-methoxyphenoxy)-3-(4-hydroxy-3-methoxyphenyl)-1,3,3a,6a-tetrahydro-4H-cyclopenta[c]furan-4-one] (Figure 5B) [49,50]. The carboxylation product retains the original phenolic hydroxyl group of curcumin. Still, the long-conjugated chain segment structure and β-diketone disappear, and the antioxidant activity is significantly reduced. Numerous structure–activity studies have shown that the direct antioxidant activity of curcumin is not only closely related to the phenolic hydroxyl group in its molecular structure but also plays a vital role in the β-diketone [25,51]. Therefore, it could be verified by the experiments of clearing MDA using Cur and restoring an acidic environment on the skin surface. Studies have shown that maintaining an acidic surface environment reduces the microbial load on the surface of skin and helps to restore the metabolism of adipose tissue [52].

The ROS produced by ultraviolet radiation attacks DNA, proteins, and biofilm systems, leading to cell damage and a series of pathological reactions, such as inflammation, gene mutations, and immune suppression [53,54]. SOD is the organism’s primary substance for scavenging free radicals. It has good antioxidation and antiaging effects on the skin, and does not easily cause allergy or irritation. SOD inhibits the peroxidation reaction of sebum, effectively fights aging, enhances skin immunity, and protects the skin from external factors such as UV rays and dust mites. By detecting the total SOD activity in the skin, it was higher in the Cur and Cur-G groups. In contrast, the total SOD activity in the Cur-L and Cur-LG groups was lower than that of the model group, considering the liposome’s slow-release properties, meaning that the drug released in a short period and was less effective for prevention or treatment. Therefore, the factors affecting the slow release of liposomes need to be verified in future experiments.

## 3. Conclusions

In conclusion, Cur-LG, compared to Cur-L, Cur-G, and Cur, enhances the antioxidant effect of curcumin. In in vitro experiments, Cur showed a solid ability to scavenge free radicals; Cur-L and Cur-G could release the drug rapidly and in large quantities. Cur-LG had a slow-release definite effect. In the experiment of preventing UV damage, Cur/Cur-L/Cur-G/Cur-LG could all effectively eliminate free radicals generated in vivo, inhibit lipid peroxidation, and promote the activity of epidermal SOD in mice; this proves that Cur can reach the site of free radical accumulation through the skin mucosa with the help of liposomes and sodium alginate gel, reduce UV damage to the skin, and prevent radiation-induced oxidative stress responses.

## 4. Materials and Methods

### 4.1. Materials

Curcumin (purity 99%) was purchased from Chengdu Pufei De Biotech Co., Ltd. (Chengdu, China). Phospholipids and cholesterol were purchased from Xi’an Jinxiang Pharmaceutical Accessories Co., Ltd. Tianjin GuangFu Chemical Research Institute provided sodium alginate (Tianjin, China). Glycerol, stearic acid, 1,1-diphenyl-2-picric acid hydrazine (DPPH), and thiobarbituric acid (TBA) were supplied by Shanghai Yuanye Biology Science and Technology Co., Ltd. (Shanghai, China). Hydrogen peroxide (H_2_O_2_) was provided by Shanghai SuYi Chemical Reagent Co., Ltd. (Shanghai, China). Trichloroacetic acid (TCA) was supplied by DAMAO Chemical Reagent Factory (Tianjin, China). The t-SOD Test kit was acquired from Jiancheng Bioengineering Institute (Nanjing, China). UVA ultraviolet lamp tube, T8 20W UVA-340 nm ± 2%, was obtained from Hongyuan Lighting. UVB ultraviolet lamp tube, G20T8E UVB-313nm, was obtained from Hongyuan Lighting. All chemical reagents were of analytical grade, and deionized distilled water was used in all experiments.

### 4.2. Animals

Healthy Kunming mice (male, 20 ± 2 g) were purchased from the Animal Experimental Center of Anhui University of Chinese Medicine (Hefei, China). All animal experiments complied with the guidelines approved by the ethics committee of Anhui University of Chinese Medicine (Hefei, China). The animals were raised under constant environmental conditions (25 ± 2 °C, 40–70% relative humidity). The animals had free access to food and sterile water.

### 4.3. Studies of the Antioxidant Activity of Curcumin In Vitro

#### 4.3.1. The Activity of Scavenging H_2_O_2_

A 0.4% solution of H_2_O_2_ was prepared with distilled water. The experiment was divided into three groups: blank group—0.6 mL phosphate-buffered solution (PBS) + 1.8 mL H_2_O_2_; sample group—0.6 mL of 0.6 mg/mL, 0.7 mg/mL, 0.8 mg/mL, 0.9 mg/mL, and 1.0 mg/mL curcumin solution + 1.8 mL H_2_O_2_; control group—0.6 mL of 1 mg/mL, 2 mg/mL, 4 mg/mL, 8 mg/mL, and 10 mg/mL curcumin solution + 1.8 mL PBS. Mixtures were evenly mixed and left to stand for 10 min to determine the absorbance at 230 nm absorbance. The scavenging rate (SE) to H_2_O_2_ was calculated as follows (1):

(1) $$\mathrm{SE}\;\left(\%\right)=\left(1-\;\frac{A_{s}{-A}_{c}}{A_{0}}\;\right)\times 100\%$$

where “A_0_” is the absorbance of the blank group, “A_s_” is the absorbance of the sample group, and “A_c_” is the absorbance of the sample control group.

#### 4.3.2. The Activity of Scavenging the DPPH Free Radical

A measure of 0.08 mg/mL DPPH was made up in absolute ethanol; the color of the solution was dark purple, and the characteristic absorption wavelength was 517 nm. When DPPH meets an antioxidant, its single electron is trapped; it lightens in color and its absorbance decreases at a wavelength of 517 nm. The decrease in absorbance level can indicate an increase in antioxidant activity [55]. The experiment was divided into three groups: blank group—2 mL PBS + 1 mL DPPH; control group—1 mL PBS + 2 mL DPPH; and other groups—1 mL + 1 mL PBS + 1mL DPPH in curcumin solution of 0.5 mg/mL, 1 mg/mL, 4 mg/mL, 6 mg/mL, and 10 mg/mL. Mixtures were left to stand in the dark for 30 min. Absorbance was determined at 517 nm. The equation for the scavenging rate (SE) of the DPPH is the same as Formula (1) above.

### 4.4. Preparation of Cur, Cur-L, Cur-G, Cur-LG

Cur was obtained by pouring 40 mg of curcumin into 10 mL of PBS and shaking well. Solution a was obtained by dissolving 40 mg of curcumin in 5 mL of PBS, and solution b was obtained by dissolving 0.3 g of phospholipids and 0.1 g of cholesterol in 3 mL of anhydrous ethanol via ultrasonication. Solutions a and b were quickly mixed and evenly mixed through ultrasonication. A constant temperature of 75 °C was maintained on a magnetic stirrer. The mixed solution was injected into 5 mL of PBS using the injection method and slowly cooled at a constant temperature for 10 minutes to obtain Cur-L. Cur-G was obtained by shaking 40 mg of curcumin and 10 mL PBS well and adding 20 mg sodium alginate, which was left to become fully swollen overnight. Cur-LG was obtained by adding 20 mg of sodium alginate to curcumin liposomes, which was left to become fully swollen overnight.

### 4.5. In Vitro Release Rate of Cur, Cur-L, Cur-G, Cur-LG

According to the existing method [56,57], the Franz diffusion cell is a device for studying the release properties of transdermal formulations in vitro. The diffusion tank was filled with PBS and the dialysis membrane was spread (MWCO: 8000–14,000 Da) on the bottle mouth to ensure there were no bubbles in the pool. The diffusion tank was placed in a magnetic stirrer. The diffusion tank was kept at a constant temperature (37 °C). A measure of 1.0 mL of PBS was added to Cur, Cur-L, Cur-G, and Cur-LG in the upper part of the diffusion cell. A measure of 2 mL of the sample was removed from the receiving cell at 5 min, 10 min, 20 min, 30 min, 1 h, 2 h, 3 h, 4 h, 5 h, 6 h, 7 h, 8 h, 9 h, 10 h, 11 h, 12 h, 24 h, 48 h, and 72 h. At the same time, 2 mL PBS was injected into the receiving cell. The absorbance of the sample was measured at a wavelength of 425 nm, and the cumulative release (R%) was plotted according to Equation (2):

(2) $$R\;\left(\%\right)=\frac{C_{n}{\times V}_{n}+\sum_{i=1}^{n-1}C_{i}{\times V}_{i}}{Q_{t}}\times 100\%$$

where “A” is the absorbance of the samples; “C” is the concentration of the drug; “C_n_” is the drug concentration of the dissolution medium; “C_i_” is the drug concentration in the sample at each sampling time point; “V_n_” and “V_i_” are the dissolution medium volume and sample volume, respectively; “Q_t_” is the theoretical drug amount.

### 4.6. Establishing Model of Photodamage Caused by UVA and UVB

One week after adaptive feeding, experiments were performed with mice. Ultraviolet light was simulated with one UVA aging test lamp and two UVB aging test lamps. Chinese Kunming mice were divided into 6 groups (N = 4): the blank group, the model group, the Cur group, the Cur-L group, the Cur-G group, and the Cur-LG group. The mice in the drug treatment group had 0.3 mL of treatment applied to their skin. The hair of skin on the backs of the mice was removed with a razor, exposing a 4 × 4 cm area to be irradiated. Each UV exposure treatment started at nine o’clock each day, with five instances a week, lasting for eight weeks. The irradiation time in the first week was 30 min, that in the second week was 60 min, that in the third week was 90 min, and those in the remaining five weeks were 120 min. Before each irradiation, the bare skin of the mouse had Cur, Cur-L, Cur-G, Cur-LG, or saline applied to it. The photos of the skin of the mice’s backs following irradiation were taken at the end of each week; the macroscopic characteristics were observed at these points as well. The scoring rules [58] for the degree of skin aging are shown in Table 1.

### 4.7. Pretreatment

After eight weeks of UV irradiation, a macroscopic skin examination was performed. The animals were weighed and the excess hair on the backs of the mice was shaved off under anesthesia. Blood samples were collected into the Eppendorf micro test tubes (EP tubes) and centrifuged (1000 r/min, 10 min) to obtain the serum. The serum was placed in heparin and trisodium citrate, and mixed evenly for 10 min after centrifugation (TG16-W, Human Xiangli Experimental Instrument Factory, Human, China) (1000 r/min, 10 min). The pellet was added inside the EP tubes; the mixture was weighed, and 2 times the volume of the high-salt phosphate buffer was added. This was dialyzed it with a dialysis bag for 24 h. The solution obtained for determining malondialdehyde (MDA) content in serum low-density lipoprotein (LDL) was applied. Another dorsal skin was washed with PBS, and the skin was removed to determine the MDA and SOD content.

### 4.8. Antioxidant Activity Measurement and MDA Content Determination

The fresh skin and liver tissue of the mice were centrifuged with a homogenizer (1500 r/min, 10 min). Mice were divided into 6 groups: blank group (1 mL tissue homogenate + 100 μL normal saline + 3 mL TBA TCA mix), model group (1 mL tissue homogenate + 100 μL normal saline + 3 mL TBA TCA mix), Cur group (1mL tissue homogenate + 100 μL Cur + 3 mL TBA TCA mix), Cur-L group (1 mL tissue homogenate + 100 μL 4 mg/mL Cur-L + 3 mL TBA TCA mixture), Cur-G group (1 mL tissue homogenate + 100 μL 4 mg/mL Cur-G + 3 mL TBA TCA mixture), Cur-LG group (1 mL tissue homogenate + 100 μL 4 mg/mL Cur-LG + 3 mL TBA TCA mixture). These were placed in a water bath at 95 °C for 40 min, rapidly cooled with water, and centrifuged (4000 r/min, 8 min). The supernatant was aspirated, and the absorbance at 532 nm was determined. The MDA content of the mouse skin and liver was calculated.

The condition of the fresh blood was observed after coagulation (1000 r/min, 10 min). A measure of 1 mL of heparin-citrate buffer was added (0.064 mol/L trisodium citrate with 5 mol/L HCL configuration 20 mL) per 100 μL serum; then, 10 mg of heparin was added, pH was adjusted to 5.04. This was mixed and placed at room temperature for 10 min, and then centrifuged (1000 r/min, 10 min). The final pH was adjusted to 5.1; the pellets were obtained and weighed. The precipitation was suspended with a high-salt phosphate buffer of 1~2 times the serum volume (pH 7.4) to dissolve the LDL precipitate. Finally, it was dialyzed for 24 h, and an LDL extract was obtained. LDL was diluted into an LDL application solution containing 25 μg/mL cholesterol with PBS. The experiment was divided into 6 groups: blank group (1 mL PBS + 1 mL LDL + 1 mL TBA + 2 mL TCA), model group (1 mL PBS + 1 mL LDL + 1 mL TBA + 2 mL TCA), Cur group (1 mL Cur + 1 mL LDL+1 mL TBA + 2 mL TCA), Cur-L group (1 mL Cur-L + 1 mL LDL+ 1 mL TBA + 2 mL TCA), Cur-G group (1 mL Cur-G + 1 mL LDL + 1 mL TBA + 2 mL TCA), and Cur-LG (1 mL Cur-LG + 1 mL LDL + 1 mL TBA + 2 mL TCA). They were placed in a bath at 95 °C for 1.5 h, cooled with running water to room temperature, and centrifuged (3000 r/min, 10 min). The absorption (A) value was measured at 532 nm, and the serum MDA content of the mice was calculated.

The mouse skin was sheared and homogenized (1500 r/min, 10 min). The SOD viability was measured according to the SOD operating instructions in the T-SOD test kit.

### 4.9. Statistical Analysis

The obtained results were expressed as mean ± standard deviation (SD). The statistical analyses were performed using GraphPad Prism Software 9.0 (GraphPad Software Corp., San Diego, CA, USA) by an analysis of variance (ANOVA) with a *t*-test. *p* < 0.05 was considered a statistically significant difference.

## Figures and Tables

**Table 1:** Scoring rules for the degree of skin aging.

Grade	Apparent Characteristic
0	No wrinkles or sagging; normal skin texture all over the body
1	Normal skin texture
2	Normal skin texture disappears
3	Shallow wrinkles
4	Few deep wrinkles and mild sagging
5	More deep wrinkles, severe wrinkles

## Data Availability

The raw data supporting the conclusions of this article will be made available by the authors on request.

## Abbreviations

UV: ultraviolet; Cur-LG: curcumin-loaded liposomes in sodium alginate gel complex preparation; Cur-L: Cur-loaded liposomes; Cur-G: Cur-loaded gel based on alginate matrix; DPPH: 1,1-Diphenyl-2-trinitrophenylhydrazine; SOD: superoxide dismutase; UVA: ultraviolet A; UVB: ultraviolet B; PBS: phosphate buffer solution; SA: sodium alginate; TBA: thiobarbituric acid; TCA: trichloroacetic acid; mL: milliliter; °C: degree Centigrade; EP tubes: Eppendorf micro test tubes; MDA: malondialdehyde; LDL: low-density lipoprotein.
